# Affective Factors in the Co-Occurrence of Personality Disorders and Substance Use Disorders

**DOI:** 10.1007/s40429-026-00719-1

**Published:** 2026-02-28

**Authors:** Andrea M. Wycoff, Timothy J. Trull

**Affiliations:** 1 Department of Psychiatry, University of Missouri, 1 Hospital Drive DC067.00, Columbia, MO 65212 USA; 2 Department of Psychological Sciences, University of Missouri, Columbia, MO 65211 USA

**Keywords:** Personality disorders, Substance use disorders, Affective processes, Affective dynamics, Comorbidity

## Abstract

**Purpose of Review:**

Personality disorders (PDs) and substance use disorders (SUDs) co-occur at high rates. Transdiagnostic mechanisms such as affective processes could improve our understanding of etiology, maintenance, and treatment of co-occurring disorders. We review the role that affective factors play in the overlap between PDs and SUDs and focus on current directions in assessment and evaluation of affective processes.

**Recent Findings:**

Recent affect-related work informing PD and SUD co-occurrence has focused on conceptualizing PDs dimensionally, testing affective processes as transdiagnostic constructs, issues in affect measurement, using ecological momentary assessment to identify proximal risk pathways, and debate on the role of affect in SUD.

**Summary:**

Affective changes can be reliably measured in daily life, and evidence supports using dimensional models of PDs compared to categorical diagnoses. Future work should build on these strengths and focus on careful translations of SUD theories to research.

## Introduction

Ample research documents high rates of co-occurrence between personality disorders (PDs) and substance use disorders (SUDs) [1–5]. Compared to individuals with one set of diagnoses or the other, individuals with both tend to experience more negative consequences and worse treatment outcomes [6, 7]. Historically, individuals with these “dual diagnoses” were considered poor candidates for treatment, and clinicians struggled to address both diagnoses simultaneously [6]. Fortunately, interventions for both PDs and SUDs have made considerable progress, and the importance of treating co-occurring conditions at the same time is being recognized [8–10]. Driven by observed heterogeneity within and overlap between disorders, transdiagnostic mechanisms such as affective processes have been highlighted as a path forward for improving our understanding of etiology, maintenance, and treatment of co-occurring diagnoses [11, 12]. We review the co-occurrence of PDs and SUDs with a focus on the role that affective factors such as negative affectivity, emotion dysregulation, negative urgency, and affective reinforcement play in their overlap.

## Prevalence of PD and SUD Co-Occurrence

Using data from the National Epidemiological Survey on Alcohol and Related Conditions (NESARC) collected between 2001 (wave 1) and 2005 (wave 2), Trull et al. [1] found that all PD diagnoses were associated with significantly increased odds of having lifetime alcohol, drug, or nicotine dependence. Individuals with any PD had five times the odds of having lifetime alcohol dependence, 12 times the odds of having lifetime drug dependence, and 3.5 times the odds of having lifetime nicotine dependence. Alcohol dependence was most prevalent among individuals with antisocial (52.1%), histrionic (49.8%), and borderline (47.4%) PDs. Drug dependence was most prevalent among individuals with histrionic (29.7%), dependent (27.3%), and antisocial (26.7%) PDs. Finally, nicotine dependence was most prevalent among individuals with antisocial (59.3%), borderline (53.9%), and dependent (53.7%) PDs.

Using the same NESARC data, Trull and colleagues [4] reported that PDs were present among 27.1% of individuals with past-year alcohol dependence, with antisocial (18.8%), borderline (12.8%), and paranoid (7.8%) PDs being the most prevalent. Among individuals with past-year drug dependence, 54.4% had any PD, with antisocial (40.2%), borderline (27.8%), and paranoid (19.7%) PDs again being the most prevalent [4].

Recent research has explored the co-occurrence of SUDs and PDs with PDs measured according to the DSM-5 Alternative Model of Personality Disorders (AMPD) [13]. For example, a recent meta-analysis of relations between the AMPD trait scores, as indexed by the Personality Inventory for DSM-5 (PID-5) [14], and symptom scores for SUDs revealed that the five domain scores of the PID-5 were all modestly and significantly related to SUD symptom counts across studies [15]. Specifically, the effect sizes of SUD symptom counts associated with PID-5 domain scores were 0.17 (Negative Affectivity), 0.31 (Detachment), 0.15 (Antagonism), 0.29 (Disinhibition), and 0.22 (Psychoticism). Thus, dimensional scores of personality pathology and SUD symptoms also show a robust association with each other, supporting the comorbidity patterns seen for DSM-5 PDs and SUD diagnoses.

## Theoretical Models of PD and SUD Co-Occurrence

Theoretical models of comorbidity suggest possible explanations for observed co-occurrence among disorders and generally include methodological and etiological reasons [16]. In the case of PD and SUD co-occurrence, specific plausible explanations have been examined in prior literature, including methodological features (e.g., the impulsivity criterion of a borderline PD [BPD] diagnosis can be met by substance use), and etiologic explanations (e.g., one disorder is a risk factor for the other) [2, 4]. Among the most compelling explanations for the co-occurrence of PDs and SUDs are that they arise from the same or overlapping etiologic processes and that features of one disorder contribute to the cause or maintenance of the other. Affective processes feature prominently in explanations of both types.

### Overlapping Affective Etiology

Affect-related traits such as neuroticism or negative affectivity could serve as underlying vulnerabilities for both PDs and SUDs. In the case of BPD, our current understanding of its etiology is that it is highly heritable, and genetic vulnerability leads to BPD in combination with adverse early childhood experiences [17]. Observed genetic markers for BPD are also present for other psychiatric disorders, highlighting an overlap in genetic vulnerability [18]. Possible transdiagnostic mechanisms linking this underlying risk to BPD include abnormalities in affective processing, which are thought to be manifestations of trait negative affectivity or neuroticism [19]. Neuroticism, or trait proneness to negative affect, is robustly associated with both PDs and SUDs [20], and could serve as a common etiologic risk factor for both sets of disorders. Indeed, Few and colleagues [21] found that genetic variation in neuroticism explained approximately 60% of the comorbidity between borderline personality traits and alcohol use disorder.

The co-occurrence between PDs and SUDs might also be partly explained by impulsivity or disinhibition. Disinhibition/impulsivity are prominent in both PDs and SUDs [22]. Although disinhibition and impulsivity are not affective constructs, negative urgency, a facet of impulsivity, ties behavioral disinhibition to negative affect. Negative urgency is characterized by the tendency to act rashly in the context of strong negative emotions and is linked to worse SUD treatment outcomes [23]. Koudys and colleagues [22] note that trait negative urgency may partly explain the comorbidity between PDs and SUDs.

### Affective Mechanisms in Directional or Reciprocal Causation

Many PDs are characterized by impairment in affective processes (e.g., high levels of negative affect, affective instability, constricted affect), which could contribute to substance use as an attempt to enhance or manage emotions. Specifically, among the traditional categorical PD diagnoses, constricted affect is present in the diagnostic criteria for schizotypal and schizoid PDs; irritability or anger in antisocial and borderline PDs; fear or anxiety in paranoid, avoidant, dependent, and borderline (fear of abandonment) PDs; and affective instability in BPD and “rapidly shifting” or shallow expression in histrionic PD [13].

The AMPD, a newer, dimensional approach to PD diagnosis, specifies impairments in the ability to experience and regulate emotional experiences as one factor in Criterion A (impairments in personality functioning), which contributes to a PD diagnosis in this dimensional framework [13]. Criterion B of the AMPD organizes pathological personality traits into five domains: negative affectivity, detachment, antagonism, disinhibition, and psychoticism. Negative affectivity of course involves several affective impairments such as lability, anxiousness, or separation insecurity, but many of the facets within other domains also implicate pathological affective processes, e.g., depressivity and restricted affect within detachment, callousness and hostility within antagonism, and impulsive behaviors driven by emotional distress within disinhibition.

Affect regulation theories of SUDs posit that individuals use substances to change their emotional states [24–27]. Motivational models of substance use describe several reasons that people use substances, including to cope with negative affect and to enhance positive affect [27, 28]. It is plausible that individuals with PD symptoms of emotional impairment might turn to substance use specifically to change their emotions. Supporting this idea, earlier cross-sectional work with college students found associations between neuroticism and alcohol coping motives [29] and that affective lability and negative affectivity were associated with alcohol problems via coping motives [30]. Longitudinal work extended these findings to show that the prospective association between cluster B PD symptoms and AUD were at least partially mediated by enhancement motives for drinking [31, 32].

More recently, using ecological momentary assessment (EMA) with individuals with BPD, we found that elevated negative affect in daily life predicted subsequent-moment alcohol use for those who endorsed coping motives more highly at baseline and that elevated positive affect predicted initiating a drinking episode for those who endorsed enhancement motives more highly at baseline [33]. With an overlapping sample of individuals where two-thirds had BPD, we also found that participants endorsed momentary enhancement motives for cigarette smoking more highly when they were also drinking alcohol compared to when they were only smoking [34]. Emerging evidence in the daily lives of individuals with BPD therefore supports the notion that individuals with PDs may be prone to substance use via emotion-modulation motives, but additional work in this area is needed.

Our summary thus far focuses on affective PD symptomology leading to problematic substance use, and past work has focused on the same causal direction whereby PD symptoms serve as a risk factor for substance use [2, 4]. However, Stetsiv and colleagues [2] pointed out that the development of PDs, which typically starts in childhood and adolescence, tends to predate initiation of substance use and escalation to substance-related problems simply due to the developmental trajectory of each type of disorder. Along these lines, although empirical evidence may implicate PD symptoms as a driver of SUD, it is important to note that substance use and related problems can exacerbate PD symptoms, in particular affective processes. For instance, theoretical models of addiction etiology emphasize the neurobiological changes that underlie progression to substance use disorder, including changes to affective processing and affective reinforcement of substance use over time [25, 35, 36].

## Emerging Trends and Current Directions

### Affective Processes as Transdiagnostic Concepts

Given considerable overlap between disorders, researchers are applying a transdiagnostic lens to examine symptomology across different diagnoses. To this end, transdiagnostic mechanisms such as affective processes have become a focus of study to improve our understanding of etiology, maintenance, and treatment of co-occurring diagnoses such as PD and SUD. Whereas negative affectivity and other specific affect-related impairments are used in PD diagnosis according to both traditional categorial diagnoses and the AMPD, negative affectivity is not explicitly included in SUD diagnostic criteria. However, recent research has supported the relevance of affective processes as transdiagnostic markers of substance use. For instance, Helle and colleagues [37] found that the affective instability symptom of BPD performed similarly to a categorical BPD diagnosis in predicting alcohol use disorder (AUD) diagnosis, number of AUD symptoms, and quantity/frequency of past-year alcohol consumption in a large epidemiological study (NESARC wave 2). Similarly, greater difficulties with emotion regulation are associated with higher frequency of substance use and more severe substance use, regardless of other psychiatric symptoms [38]. Two recent meta-analyses report robust associations between difficulties with emotion regulation and SUD diagnosis [39] and between emotion dysregulation and many addictive behaviors including cannabis problems, cannabis severity, gaming severity, gambling severity, gambling problems, alcohol problems, alcohol severity, and nicotine dependence severity [12].

Research on transdiagnostic constructs like emotion (dys)regulation can inform etiology and more efficient and effective interventions for co-occurring disorders [40]. Notably, in a systematic review, Sloan and colleagues [11] found improvements in emotion dysregulation following psychological treatment for SUD, BPD, and other disorders, which offers support for the idea that emotion dysregulation is a clinically significant transdiagnostic construct. Preliminary evidence also highlights specific emotion regulation strategies as potential mediators of outcomes in the context of a transdiagnostic treatment [41]. Emotion dysregulation and other affective processes may be particularly useful treatment targets for individuals with PD(s) and SUD(s).

### Intensive Longitudinal Designs

To precisely measure affective processes that may be relevant to SUDs, it is necessary to move beyond static, cross-sectional measures. Specifically, cross-sectional measures of affective processes require participants to accurately characterize affective intensities and patterns over some period of time. However, it is well-known that such assessments are subject to a number of heuristic biases like primacy/recency and peak-end [42], and the accuracy of momentary reports is higher than retrospective reports of events, behaviors, and experiences [43, 44].

Therefore, many researchers use methods like ecological momentary assessment (EMA; a.k.a ambulatory assessment) [45, 46] to better assess psychological processes like affect and affective dynamics. The data gathered through EMA are often referred to as intensive longitudinal data (ILD). Because EMA involves multiple assessments over time, it is well-suited to focus on within-individual processes. For example, the experience of anxiety is dynamic and may ebb and flow over time, often alongside contextual or environmental factors. Traditional cross-sectional assessment requires individuals to somehow characterize their symptoms by aggregating in some unspecified way over extended periods of time and requires some degree of retrospection. In contrast, EMA provides repeated assessments of momentary experiences (e.g., “within the last 15 minutes”), minimizing retrospective biases and reliance on memory heuristics. EMA captures slices of these processes in real or near-real time, allowing an evaluation of not only affective processes (e.g., how much one’s anxiety changes within and across days) but also potential internal and external influences on these processes. Thus, EMA adds a needed time dimension to the assessment of affective and other psychological processes. Finally, due to the collection of data during individuals’ daily lives, the ecological and external validity of these assessments, by definition, exceeds that of more traditional approaches.

Important affects and moods like depression, anxiety, and anger that are characteristic of PDs (and potentially SUDs) and assessed using multiple momentary assessments in EMA can therefore be further evaluated in terms of dynamic patterns that require assessments over time. For example, in an EMA study of 74 outpatients diagnosed with borderline personality disorder, Jahng et al. [47] found that while mean levels of affect did not distinguish those outpatients that drank alcohol from those that did not, those BPD outpatients that drank alcohol were distinguished by more affective variability within day in negative affect and fear/anxiety and day-to-day variability in negative affect, fear/anxiety, and sadness.

More recently, researchers have used EMA to examine affect and aspects of dimensional models of PDs in daily life. For instance, among adolescent girls, Kaurin and colleagues [48] found that maladaptive personality traits were associated with EMA-assessed boredom, interpersonal tension, and negative affect. In multiple samples enriched for PD features, Vize and colleagues [49] found proximal reciprocal associations between negative emotions and interpersonal conflict. Given its suitability for studying how affective processes, PD dynamics, and substance use events unfold in individuals’ natural environments, future work should use EMA to integrate these lines of research to better understand the role of affect in the overlap of PDs and SUDs.

### Measurement of Affective Processes

The term “affective processes” is broad and encompasses many constructs that are related but distinct and have distinct measurement needs. Negative affectivity and disinhibition, for example, are two of the five domains of Criterion B of the AMPD and are composed of several affective facets including (for negative affectivity) emotional lability, anxiousness, separation insecurity, hostility, depressivity, and restricted affectivity, and (for disinhibition) impulsivity including urgency in the context of emotional distress [13]. Recent studies support the measurement of negative affectivity and disinhibition as trait-level constructs in terms of outperforming categorical PD diagnoses in the prediction of clinical outcomes [50]. For instance, disinhibition predicted significant incremental variance in problematic substance use above and beyond categorical diagnoses [51]. In addition, negative affectivity outperformed categorical PD diagnoses in prospectively predicting naturalistically-observed negative affect one year later, with specific facets of anxiousness, emotional lability, and separation insecurity significantly correlated with observed negative affect [52]. Thus, measuring affect-related personality domains and facets from the AMPD framework could improve our understanding of affect’s role in the co-occurrence of PDs and SUDs to the extent that it captures meaningful variation in negative affect and substance use that might be missed by categorical PD diagnoses.

Emotion dysregulation has been defined as a specific process consisting of multiple components including sensitivity to emotional stimuli, heightened and labile negative affect, a lack of or ineffective emotion regulation strategies, and consequences of emotion dysregulation [53]. There are already established measures of emotion regulation strategies [54, 55], but we saw a need for a screening tool that assesses the other components of emotion dysregulation and is developed outside of the context of BPD. Accordingly, we recently developed the 12-item Brief Emotion Dysregulation Scale (BEDS) [56] to measure emotion dysregulation, specifically, sensitivity to emotional stimuli, emotional lability, and consequences of emotion dysregulation. We developed it recognizing that emotion dysregulation is a transdiagnostic construct, and we showed its correlations with substance use and personality pathology. The BEDS lability subscale was positively associated with scores on the Alcohol Use Disorders Identification Test [57] and Drug Use Disorders Identification Test [58]; BEDS lability and consequences were positively associated with all PID-5-BF factors [59], PAI-BOR total score and all subscales [60]; BEDS sensitivity was positively associated with PID-5 negative affectivity; and BEDS sensitivity, lability, and consequences were all significantly associated with UPPS-P negative urgency [61]. Thus, the BEDS is a concise tool for screening emotion dysregulation outside of the context of one specific disorder or set of disorders and, given its associations with symptoms of both PDs and SUDs, may be especially useful in examinations of their co-occurrence.

In addition to measuring affect and emotion dysregulation at the trait level, affective processes have long been understood to be dynamic and change over time [62, 63]. Trull et al. [62] outlined how EMA data can be used to characterize affective processes, including affective instability (via mean-squared successive differences; MSSDs), acute affective changes (via probability of acute change; PAC), affective inertia (via within person autocorrelation), and affect differentiation (through in-the-moment intraclass correlation coefficients within person). Affective instability combines concepts of affect variability with temporal order to provide unique information about affect’s movement across some period of time. Affective instability can be assessed at the trait level, as in a BPD diagnosis or measures of lability, but as mentioned above, retrospective measurement is subject to recall biases and inaccuracies. Repeated momentary assessments of affect via EMA allow for temporally specific and fine-grained examinations of the proximal associations between affective instability and other constructs of interest, like personality functioning or substance use.

Measuring affect, and especially affective changes, via EMA requires reliable measurements, but it remains uncommon to evaluate and report the reliability of measures used in EMA [64]. To remedy this problem, our group recently evaluated the reliability of the Positive and Negative Affect Schedule—Expanded Form (PANAS-X) [65] in four EMA studies of adults, most of whom were receiving outpatient psychiatric treatment and many of whom met criteria for BPD [66]. Using generalizability theory [67] and SEM frameworks [68], we found that the positive affect, negative affect, fear, sadness, and hostility scales of the PANAS-X can reliably capture not only between-person differences but also within-person change, which is crucial for EMA research seeking to understand how affective processes unfold and relate to other symptoms of interest.

### Debate on the Role of Affect in SUD

Currently, there is some debate in the field about what role affect plays in SUDs. This debate is specifically related to negative affect and stems primarily from the EMA literature on affect as a precursor to alcohol use. As we noted above, several theoretical models of addiction posit that individuals use substances to change their affective states [24, 25, 27, 69]. A reasonable hypothesis follows, which is that individuals who drink alcohol should be more likely to drink when negative affect is higher than usual, especially if they say they tend to drink to cope with negative emotions. Many researchers have used EMA to examine this question with mixed findings, culminating in a recent meta-analysis of individual participant data by Dora and colleagues [70], which found that negative affect was not related to alcohol use at the daily level, not even among participants who highly endorsed drinking-to-cope motives at baseline. This finding could be interpreted as evidence that our theories are wrong, but our view is that this finding should push addiction researchers to more carefully consider how to operationalize affect regulation theories in daily life.

For example, Conger [69] wrote about the drive reduction properties of alcohol in rats with respect to their approach tendency (to obtain food) versus avoidance tendency (to avoid an electric shock) but is frequently cited as tension reduction theory in terms of alcohol reducing stress/negative affect. Motivational models posit that individuals use substances to change their emotional states but specify intermediate constructs such as expectancies and motives that link emotional states to actual substance use [27, 28]. Koob and Volkow [25] discuss increased negative emotional states and negative reinforcement mechanisms only once an individual has progressed to stages of severe SUD, including experiencing withdrawal. Thus, to more closely evaluate these theories using EMA, we would need to focus on fear and disinhibited behavior under intoxication [69], measure expectancies and motives at the momentary level rather than at baseline [27, 28, 71–73], and recruit individuals with severe SUDs who experience withdrawal [25]. More careful operationalizations of affect-related SUD theories (and augmentation of our theories if warranted) is a necessary precursor to more thorough investigations of how affect serves as a link between PD and SUD expression.

## Conclusions

PDs and SUDs co-occur at high rates. Theories and recent empirical evidence support focusing on transdiagnostic affective processes like emotion (dys)regulation to better understand the etiology, maintenance, and treatment of co-occurring PDs and SUDs. Collectively, recent advances in conceptualizing PDs dimensionally rather than categorically, using intensive longitudinal designs like EMA to elucidate proximal risk pathways, and reliable measurements of affect and affective processes can move the field forward in understanding how affect contributes to PD and SUD overlap. Recent debate in the SUD literature on how affect is related to substance use highlights a need for more carefully considered and theoretically-grounded research on daily-life affective processes surrounding substance use, which will lay a better foundation for examining the role of affect in PD and SUD co-occurrence.

## Key References

- Stetsiv K, McNamara IA, Nance M, Carpenter RW. The co-occurrence of personality disorders and substance use disorders. Current Psychiatry Reports. 2023;25(11):545–54. doi: 10.1007/s11920-023-01452-6. 
  - Recent review of the prevalence rates and theoretical explanations of the co-occurrence of personality disorders and substance use disorders.
- Stellern J, Xiao KB, Grennell E, Sanches M, Gowin JL, Sloan ME. Emotion regulation in substance use disorders: a systematic review and meta-analysis. Addiction. 2023;118(1):30–47. doi: 10.1111/add.16001. 
  - Comprehensive review of 22 studies to examine whether individuals with substance use disorders have deficits in emotion regulation compared to controls.
- Lincoln TM, Schulze L, Renneberg B. The role of emotion regulation in the characterization, development and treatment of psychopathology. Nature Reviews Psychology. 2022;1:272–86. doi: 10.1038/s44159-022-00040-4. 
  - Review of theoretical models of emotion regulation, associations between specific emotion regulation difficulties and a range of psychopathology, and transdiagnostic interventions that emphasize emotion regulation skills.
- Vize CE, Ringwald WR, Scott LN, Kamarck TW, Pilkonis PA, Wright AGC. Evidence for a Vicious Socioemotional Cycle of Negative Emotions and Interpersonal Conflict. Journal of Consulting and Clinical Psychology. 2024;92(8):479–92. doi: 10.1037/ccp0000891. 
  - Nice example of examining dynamic associations between personality functioning and negative affect in daily life.
- Sharp C, Miller JD. Head-to-Head Comparisons of Diagnostic and Statistical Manual of Mental Disorders, Fifth Edition, Section II and Section III Personality Disorder in Predicting Clinical Outcomes. Personality Disorders: Theory, Research, and Treatment. 2024;15(5):275–81. doi: 10.1037/per0000691. 
  - Introduction to a special issue including summaries of articles in the issue that compare categorical and dimensional personality disorder diagnoses in their ability to predict clinical outcomes.
- Wycoff AM, Griffin SA, Helle AC, Haney AM, Watts AL, Trull TJ. The Brief Emotion Dysregulation Scale: Development, Preliminary Validation, and Recommendations for Use. Assessment. 2024;31(2):335–49. doi: 10.1177/10731911231161800.
  - Measure development for a brief, transdiagnostic measure of emotion dysregulation including sensitivity, lability, and consequences, and its associations with substance use and personality disorder validators.
- Haney AM, Fleming MN, Wycoff AM, Griffin SA, Trull TJ. Measuring affect in daily life: A multilevel psychometric evaluation of the PANAS-X across four ecological momentary assessment samples. Psychol Assess. 2023;35(6):469–83. doi: 10.1037/pas0001231. 
  - Examination of the reliability of the PANAS-X in four EMA studies, demonstrating that the PANAS-X can reliabily detect affective changes in daily life.
- Dora J, Piccirillo M, Foster KT, Arbeau K, Armeli S, Auriacombe M, et al. The daily association between affect and alcohol use: A meta-analysis of individual participant data. Psychological Bulletin. 2023;149(1–2):1–24. doi: 10.1037/bul0000387.
  - Meta-analysis of individual participant data from 69 studies demonstrating no association between negative affect and same-day alcohol use.
- Wycoff AM, Trull TJ. Affective reinforcement from simultaneous versus single use of alcohol and cannabis. Drug and Alcohol Dependence. 2025;270:112612. doi: 10.1016/j.drugalcdep.2025.112612. 
  - Example of using EMA to advance the literature on affect’s role in addiction by measuring affect regulation motives for alcohol and cannabis use at the momentary level.

## Data Availability

No datasets were generated or analysed during the current study.
